# 

**Published:** 1886-10-20

**Authors:** 


### SUBSCRIBERS IN ARREARS.

There are certain of our subscribers whose actions imply that they think we can publish a journal and mail it to them for a long while without compensation. We suppose it is in most instances the result of carelessness, which we are sorry to have to say is a too common fault with Doctors. This ought not to be so. Of all men in the world the physician should be true to his pecuniary obligations. To those who have been punctual in remitting their dues, we are very thankful, and we trust, now that the year is drawing to a close, that all who are in arrears will think of us as a friend who has faithfully fulfilled his part of the contract in furnishing the RECORD, and who now needs that remuneration for his services due from the other party.

MANAGING EDITOR.

### RECEIPTS.

1885.—W. H. Peek, R. C. Waters, James M. Barton.

1886.—G. W. Wright, to July; R. M. Savage, L. W. Coleman, C. J. Burroughs, C. M. Bold, M. P. Deadwyler, L. H. Cartledge, John Elsner.

1887.—M. M. More, to September; H. H. Whitaker, to October; J. R. Reeve, to April; T. J. Lee, to February; J. S. Boyce.

1888.—T. S. Jones, to July.

